# Restimulation could stop status epilepticus after electroconvulsive therapy: 2 case reports

**DOI:** 10.3389/fpsyt.2025.1576374

**Published:** 2025-05-29

**Authors:** Michael Pinchuk, Kaat Hebbrecht, Pascal Sienaert, Elizabet Boon, Filip Bouckaert

**Affiliations:** ^1^ University Psychiatric Centre KU Leuven, Leuven, Belgium; ^2^ KU Leuven, Department of Neurosciences, Research Group Psychiatry, Neuropsychiatry, Academic Center for ECT and Neuromodulation (AcCENT), University Psychiatric Center KU Leuven, Kortenberg, Belgium; ^3^ KU Leuven, Leuven Brain Institute, Department of Neurosciences, Neuropsychiatry, B-3000, Leuven, Belgium; Geriatric Psychiatry, University Psychiatric Centre KU Leuven, Leuven, Belgium

**Keywords:** electroconvulsive therapy, status epilepticus, prolonged seizure, mechanism, complication, anticonvulsant hypothesis, restimulation

## Abstract

**Background:**

Electroconvulsive therapy (ECT) is an effective treatment for severe depression, mania, psychosis and catatonia. While seizures are considered essential for the therapeutic effect of ECT, it concurrently has an anticonvulsant effect which plays a role in its mechanism of action. This property has also prompted the use of ECT in managing status epilepticus (SE).

**Case Presentation:**

We report two distinct cases of prolonged seizures during ECT that persisted for more than 5 min despite administration of propofol and lorazepam, ultimately meeting criteria for status epilepticus (SE). The first case involved an 80-year old woman with severe psychotic depression starting ECT, while the second case involved a 30-year old man receiving maintenance ECT for difficult-to-treat schizophrenic psychosis. In both cases, SE was promptly terminated by restimulation, defined as an additional stimulus delivered within the same ECT session. After epilepsy and intracranial pathology were ruled out, ECT was safely resumed in both patients after switching from etomidate to propofol induction.

**Conclusion:**

Status epilepticus after ECT can be resolved by restimulation when standard interventions are unsuccessful, thereby avoiding potential neurological complications. We provide an overview of the mechanism and current clinical evidence supporting this strategy, and propose an amended clinical practice protocol for SE after ECT.

## 1 Introduction

Electroconvulsive therapy (ECT) is an effective treatment for difficult-to-treat depression, particularly in older patients and when psychotic features are present (1). It is also a second-line treatment for clozapine-resistant psychosis (2). While there is an abundance of evidence for the efficacy and safety of ECT (3), the mechanism of action remains unresolved. ECT was developed in 1938 as a safe way to elicit a seizure, as it was believed that seizures counteracted psychosis (4). The three currently most accepted hypotheses still stem from the assumption that seizures are directly involved in the therapeutic effect of ECT. The generalized seizure hypothesis posits that the therapeutic effect of ECT is dependent on the elicitation of generalized seizures (5), while the combined anatomical-ictal hypothesis suggests that therapeutic effect is driven by seizure activity in the limbic system which induces neurotrophic effects through brain derived neurotrophic factor (BDNF) (6, 7). The anticonvulsant hypothesis suggests that the therapeutic effect of ECT originates from an increased inhibitory GABA-ergic neurotransmission, as the seizure threshold often rises during a course of ECT (8, 9). This phenomenon has facilitated the use of ECT in status epilepticus (SE) (10). Status epilepticus is defined by the International League Against Epilepsy (ILAE) as a generalized seizure lasting more than 5 min, which is considered a practical time point for initiating treatment, or more than 30 min, beyond which significant risk of long-term neuronal injury and functional deficits arise (11).

Prolonged seizures after ECT are seizures of >180 seconds occurring in 1-2% of ECT courses (12) and are typically managed by intravenous anesthesia or benzodiazepines (13). However, in rare cases these interventions are ineffective leading to SE (14). Tardive seizures after ECT, meaning seizure activity after termination of the therapeutic seizure, can also occur (15). Managing SE poses significant clinical challenges. Evidence guiding interventions is limited and entails general intensive care, antiepileptic drugs and treatment of underlying pathology (11, 16). We illustrate the paradoxical relationship between seizure and ECT by presenting two cases where SE following ECT was promptly managed by restimulation.

## 2 Case presentation

### 2.1 Case A

#### 2.1.1 Patient information

Ms. A, an 80-year-old woman, was admitted for severe depression with psychotic features. She had no prior psychiatric or neurological history, and no known family history of depression or epilepsy. She had a history of breast cancer with bone metastasis diagnosed in the previous year. She had been treated with escitalopram 15 mg and mirtazapine 15 mg for three months before admission without any clinical improvement. Further medication consisted of letrozole 2.5 mg.

#### 2.1.2 Clinical findings and diagnostic assessment

The patient exhibited depressed mood, anhedonia, cognitive impairment, and psychotic features such as nihilistic delusions and paranoid behavior. Her Montgomery-Åsberg Depression Rating Scale (MADRS) (17) score was 40/60, and her CORE score was 15, suggestive of a melancholic depression (18). Upon admission, olanzapine 5 mg was added to the regimen which showed no effect after the first week. Given the severity of her symptoms, ECT was advised and started after informed consent by proxy was granted by the patient’s family. Pre-ECT evaluations, including EKG and laboratory tests, were unremarkable.

#### 2.1.3 Therapeutic intervention

**Figure 1:**
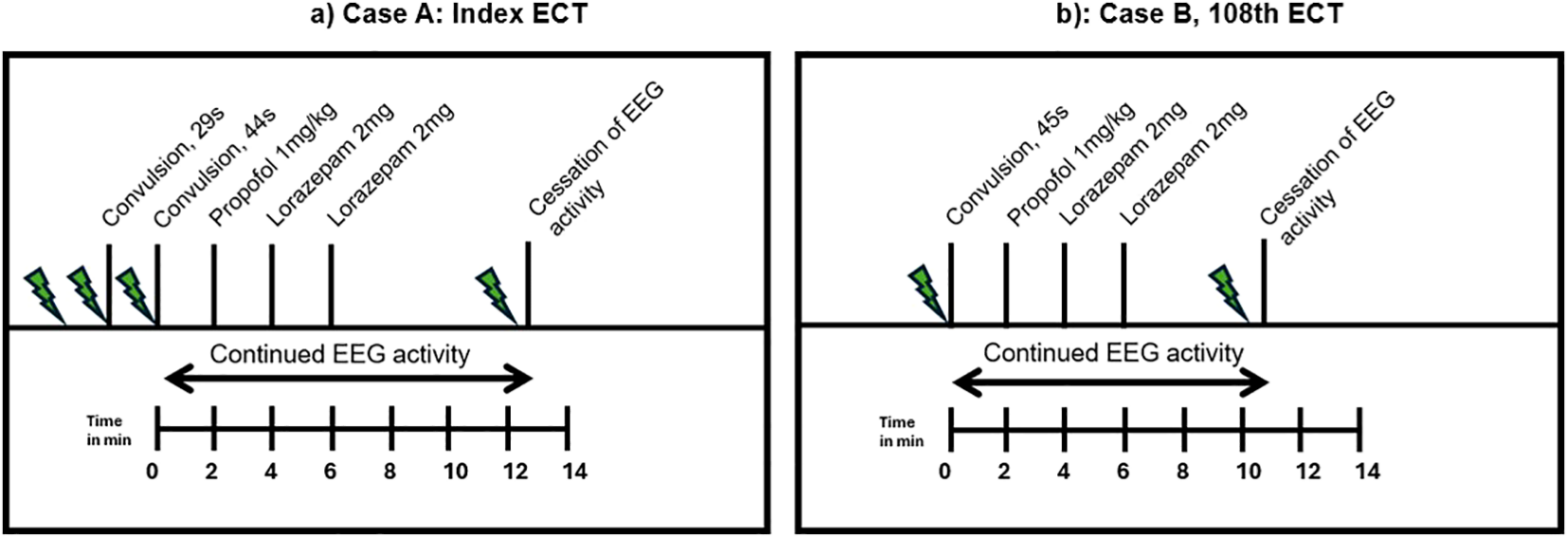
**(a)** Case A, Index ECT. **(b)** Case B, 108^th^ ECT.

**Figure 2:**
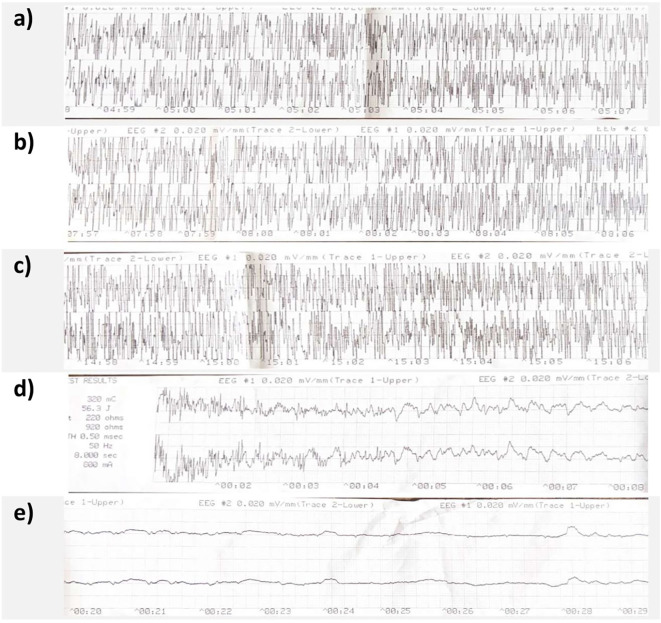
Case A's MECTA 2-channel EEG. Panel (**a–c**) show continued spike and wave activity at 5 min, 8 min and 15 min respectively. Panel **(d)** shows restimulation at 15 min with termination of clear spike and wave activity, panel **(e)** shows clear postictal suppression 20 seconds after restimulation.

Right unilateral ECT twice a week was started using a square-wave, brief-pulse, constant-current device (MECTA SR1-5000Q; Lake Oswego, Oregon). **Figure 1a** shows a timeline of the index ECT. Anesthesia consisted of etomidate 12 mg, succinylcholine 35 mg and 100% oxygen. The seizure threshold was established by empirical titration (see **Table 1**). The second titration step resulted in a threshold seizure, followed by a therapeutic stimulus at 6 times seizure threshold. The following seizure exceeded 2 min on electroencephalogram (EEG). Per hospital protocol (see **Table 2a**), propofol 60 mg was administered, followed by lorazepam 2 mg at 4 min and an additional 2 mg at 6 min. Despite these interventions, EEG showed sustained spike and wave activity consistent with SE (**Figure 2**). The clinical team decided to administer another stimulus using the same parameters applied 15 min after the first. After restimulation, EEG monitoring showed immediate cessation of seizure activity, followed by postictal suppression.

**Table 1:** Stimulus parameters for Case A and B.

Stimulus Nr.	Stimulus type	Electrode position	Pulsewidth	Pulse frequency	Train	Charge
Case A						
1	Titration	RUL	0.5ms	20Hz	1.75s	28mC
2	Titration	RUL	0.5ms	20Hz	3.25s	52mC
3	Therapeutic	RUL	0.5ms	50Hz	8s	320mC
4	Termination	RUL	0.5ms	50Hz	8s	320mC
Case B						
1	Therapeutic	BT	0.5ms	20Hz	3.75	60mC
2	Termination	BT	0.5ms	20Hz	3.75	60mC

RUL, Right unilateral electrode position; BT, Bitemporal electrode position.

**Table 2:** Protocol to manage prolonged seizures after ECT.

a. Current protocol at University Psychiatric Center KU Leuven	
Duration of convulsion	Action
1 min 30 seconds	Prepare propofol 1 mg/kg
2 min	Administer propofol 1 mg/kg
4 min	Administer lorazepam 2 mg
6 min	Administer lorazepam 2 mg
b. Proposed amendment based on current report	
10–15 min	Consider restimulation
15 min	Transfer to expert acute neurological care
Follow up:	· 24h in-hospital monitoring · Neurological consult with 24-channel EEG to rule out epilepsy · Consider intracranial imaging to rule out intracranial pathology · Consider stopping medication that lowers seizure threshold · Switch to propofol induction if ECT would be resumed

#### 2.1.4 Follow-up and outcomes

Post-ECT, Ms. A was closely monitored and her vital signs and neurological status remained stable. A neurologist (EB) evaluated the patient, and subsequent 24-channel EEG showed no epileptic activity. A CT ruled out intracranial pathology, including breast cancer metastasis. After weighing risks and benefits with the patient’s family, ECT was resumed using propofol for induction. The patient tolerated subsequent sessions without complications. After 8 sessions her MADRS score decreased to 4 and CORE score to 0, indicating remission. She was discharged with maintenance ECT and continued to do well at follow-up after 6 months.

#### 2.1.5 Patient perspective

Ms. A recalls little about her depressive symptoms and often wonders what she was doing in the months before her hospitalization. She felt well-informed about side effects before and during treatment. As she was unconscious during the status epilepticus and still severely depressed afterward, she has limited recollection of discussions about the events. Her son, who was also informed, felt he received adequate explanations regarding what happened. Both Ms. A and her family emphasize that they mainly remember the rapid and complete remission of depression after ECT sessions. They also emphasized the importance of the kindness and warmth of the ECT team as a key aspect of her care. At the time of writing maintenance ECT was discontinued and Ms. A remains in remission.

### 2.2 Case B

#### 2.2.1 Patient information

Mr. B was a 30-year old male diagnosed with schizophrenia, showing first symptoms of disorganization and paranoid delusion at 17 with severe impact on his functioning. Before admission, he was treated with Amisulpride 400 mg, olanzapine 10 mg and paliperidone long acting injection 150 mg with little improvement in functioning, which lead to the diagnosis of difficult-to-treat schizophrenia. Physically he was diagnosed with Juvenile Polyposis Syndrome at 11, for which he received a total colectomy. His current admission started several years before the event for non-suicidal self-injurious behavior and catatonia.

#### 2.2.2 Clinical findings and diagnostic assessment

On admission, Mr. B showed mannerisms, stereotypical behaviors, autonomic instability, perseverations and non-suicidal self-injurious behavior, particularly severe scratching, leading to diagnosis of schizophrenia with catatonia. A CT brain and extensive blood work showed no abnormalities, inferring no organic etiologies of catatonia. Alongside clorazepate 15 mg 3x/day, clozapine 200 mg was initiated and initially provided partial improvement. However, residual stereotypical behaviors and scratching persisted. Clozapine was eventually discontinued because of recurrent gastrointestinal obstruction, which was considered a side effect aggravated by Juvenile Polyposis Syndrome and colectomy. After multidisciplinary discussion and pre-ECT evaluations, ECT was advised and informed consent by proxy was obtained from his family.

#### 2.2.3 Therapeutic intervention

Bitemporal ECT for catatonia in schizophrenia was started with good effect on catatonic symptoms and non-suicidal self-injury. The reduction in symptoms led to an improvement of activities of daily living on the ward. Reduction to biweekly ECT led to an increase in catatonic symptoms, after which weekly ECT was continued. Aside from clorazepate 15 mg 3x/day, he was on aripiprazole 15 mg and clotiapine 20 mg 3x/day. Furthermore he received lorazepam 2.5 mg as needed. ECT was continued with important clinical improvement for 107 sessions. During his weekly maintenance ECT treatment, the patient had a prolonged seizure on session 100 necessitating propofol with successful termination of the seizure.

On the 108^th^ ECT session, he received 16 mg etomidate and 50 mg succinylcholine for induction. **Figure 1b** shows a timeline of this ECT session. Therapeutic stimulus was given with the same parameters as previous stimulations (see **Table 1**), and motor convulsions terminated at 45 seconds. He showed epileptic activity on EEG for more than 2 min, after which the same protocol as above was followed (see also **Table 2a**). EEG showed continued epileptic activity after 8 min. The clinical team decided to administer 8 mg etomidate and 50 mg succinylcholine and stimulate the patient 10 min 43s after the first stimulation with the same dose as the therapeutic stimulus. A 25-second convulsion ensued, after which EEG monitoring showed immediate cessation of seizure activity and postictal suppression.

#### 2.2.4 Follow-up and outcomes

The patient was closely monitored for 24 hours and received a neurological follow up consultation with 24-channel EEG which showed no epileptic activity. After careful consideration, imaging was not performed since there was no indication that the patient had any structural brain abnormality. Discharge of residential hospitalization was already being planned in the time leading up to this event, but was only possible due to continued improvement with weekly ECT. Therefore weekly ECT was resumed with propofol induction. After no prolonged seizures or other complications were reported in the next month, the patient was discharged from residential hospitalization, while continuing weekly maintenance ECT.

#### 2.2.5 Patient perspective

Communicating with Mr. B remained challenging even after symptom improvement with ECT, making it difficult to fully understand his personal experience of the treatment. However, given the severity of his symptoms, it was evident that he endured significant suffering. As he had no recollection of the SE, he expressed no concern about its implications. His father was more worried about potential cognitive side effects of ECT than the prolonged seizures.

With continued maintenance ECT sessions, Mr. B showed noticeable improvement in paranoid delusions, stereotypical behaviors, excessive scratching, and disorganization, allowing for better engagement in activities of daily living. One month after the SE, he was discharged from the hospital after several years of inpatient care and transitioned to a psychiatric care home. However, he continues to experience disorganization and is still receiving maintenance ECT at the time of writing.

## 3 Discussion

### 3.1 Mechanism

The anticonvulsant effects of ECT have long been recognized, giving rise to the anticonvulsant hypothesis of its mechanism of action. This hypothesis states that increased inhibitory GABA-ergic neurotransmission is necessary for the therapeutic effect of ECT (8, 9). As genetic deficits of GABA-ergic metabolism lead to epileptic syndromes and many GABA-agonists are anticonvulsants, we know that GABA plays a central role in seizures. Furthermore, GABA might stimulate neuroplasticity (19). During the course of ECT, seizure duration decreases while seizure threshold increases (20), which could be due to increased levels of GABA, GABA-receptor activity and GABA-ergic interneurons (21–24) and may be linked to neuroplastic effects of ECT (20). Postictal suppression, seen at the end of an ECT-induced seizure on EEG, could be the expression of an increased postictal inhibitory process and appears to be a useful predictor of clinical outcome of depression (25, 26).

The anticonvulsant hypothesis has provided a theoretical basis for the use of ECT as a treatment for SE. A recent scoping review describes 28 patients with refractory or super-refractory SE that received ECT, all of which resulted in SE resolution, with clinical improvement reported in 20 patients (10). ECT is classified at a GRADE D/Oxford level 4 evidence for treatment of SE (10, 27) and is mentioned in 5 clinical practice guidelines as alternative therapy for specific cases of refractory and super refractory SE (28). The limited evidence supporting these clinical recommendations highlights the relevance of our report.

### 3.2 Current findings and clinical practice

The cases described in our study demonstrate that administering an additional ECT stimulus can effectively terminate SE when conventional treatments fail. This approach is theoretically grounded in the anticonvulsive hypothesis and in clinical evidence showing that ECT is an effective treatment for refractory SE. Although propofol induction prevented prolonged seizures in subsequent ECT sessions and is usually sufficient to terminate prolonged seizures, it was ineffective in these two cases. Similarly, the ensuing doses of lorazepam were insufficient. The temporal relationship between the stimulus and the swift cessation of the seizure reinforces this hypothesis. Additionally, this approach was validated in two patients. It should be noted that in case B, we used etomidate in preparation for the terminating stimulus instead of propofol. Although this strengthens the hypothesis that the seizure stopped because of the stimulus and not due to additional anesthesia, propofol bears preference due to its stronger anticonvulsive properties (29).

Our cases can be considered both a prolonged seizure and SE, explaining why terminology in literature of abnormal seizures after ECT is heterogenous. We consider all prolonged or tardive seizures with >5 min of generalized seizure activity, or all partial and absence seizures >10 min as status epilepticus, based on the ILAE classification (11). In a search of the literature we found 35 cases of status epilepticus after ECT meeting these criteria (14, 30–63) (see **Supplementary Materials** for search method). Only two of these describe restimulation to terminate SE. Hazimeh et al. (28) describe a convulsion starting 11 min after ECT which was initially managed with midazolam and propofol. When convulsions resumed and propofol had no effect, the convulsion ceased after the second stimulus. However, more convulsions followed after 5 min and SE was not resolved by this intervention. Goh et al. (37) describe a prolonged seizure of more than 12 min, constituting SE, which was terminated completely by a second stimulus. In these two cases described in the literature, the eliciting stimulus was the seizure threshold, and both terminating stimuli used were six times seizure threshold equivalent to a therapeutic stimulus. Conversely, in our cases the eliciting stimulus was therapeutic and both terminating stimuli were of the same dose, suggesting the dose of the terminating stimulus is not a critical factor in the mechanism of seizure termination. It has indeed been shown that seizure duration decreases between the first and second treatment, while the relationship between stimulus dosage and seizure duration is less straightforward (64). It is also known that an effective ECT session results in an immediate and substantial surge of GABA (22). The finding that the second stimulus is more effective than the administered medication could be explained by the electrical stimulus provoking an excitatory wave immediately followed by a massive outpouring of GABA and other inhibitory neurotransmitters (19). However, if the eliciting stimulus dose is the seizure threshold, we would recommend restimulating with a therapeutic stimulus.

Prolonged seizure, SE, and tardive seizures after ECT share risk factors that lower seizure threshold, which should be considered and mitigated following an abnormal seizure after ECT:

- Medication: clozapine (59), lithium (65), bupropion, antibiotics, theophylline (15).
 Seizure risk may increase after recent tapering of antiepileptics or benzodiazepines (15).
- ECT delivery: first sessions (15), multiple monitored ECT (30).
- Anesthesia: etomidate (29), hyperventilation, anesthetic-ECT time interval (66).
- Patient specific factors: younger age (65), women (67), height (64), intracranial pathology like brain metastasis (42), although ECT can remain safe in these patients (68).

Based on these findings, we propose amendments to the protocol used to manage prolonged seizure after ECT (See **Table 2b**). We suggest considering restimulation after all other treatments have failed. All 4 available reports restimulated 10–15 min after the eliciting stimulus, and it should be considered before 30 min of SE as risk of neurological complications is significant by this time (11). Additionally, we suggest minimizing risk factors for prolonged seizures like intracranial pathology and medication before considering resumption of ECT. Finally, we propose to resume ECT using propofol induction as this raises the seizure threshold (29). Further research is necessary to bolster the evidence for these recommendations, although we report that both of our patients were able to continue ECT without further complications and with important clinical benefit.

### 3.3 Limitations

Aside from publication bias and possible overinterpretation which are limitations inherent to case series (69), a limitation of our report is that we do not have 24-channel EEG data of the events themselves as patients in our center are monitored through the MECTA 2-channel EEG during ECT. Difficulty in diagnosing SE, particularly non-convulsive SE, has been noted in previous reports, as EEG slowing after ECT is a physiological phenomenon (70–72). Additionally, while ECT has been safely used in SE, our report is not able to offer a comparative analysis of the tolerance of this intervention. However, we offer suggestions on how to manage follow-up.

### 3.4 Conclusion and implications

This report suggests that status epilepticus after ECT can be safely treated by restimulation, avoiding a longer seizure and potential severe neurological complications. Our report describes the theoretical foundations and acknowledges two previous reports with this finding, leading us to believe this strategy is warranted if this rare complication arises. After risk factors were determined, anesthesia was switched to propofol and both patients resumed ECT without complications. Further research would have to validate this strategy which might offer a safe and effective way to address SE when other treatments fail.

## Data Availability

The original contributions presented in the study are included in the article/**Supplementary Material**. Further inquiries can be directed to the corresponding author/s.

## References

[1] Van Diermen L Van Den Ameele S Kamperman AM Sabbe BCG Vermeulen T Schrijvers D . Prediction of electroconvulsive therapy response and remission in major depression: Meta-analysis. Br J Psychiatry. (2018) 212:71–80. doi: 10.1192/bjp.2017.28 29436330

[2] Grover S Sahoo S Rabha A Koirala R . ECT in schizophrenia: A review of the evidence. Acta Neuropsychiatr. (2019) 31:115–27. doi: 10.1017/neu.2018.32 30501675

[3] The UK ECT review . Efficacy and safety of electroconvulsive therapy in depressive disorders: a systematic review and meta-analysis. Lancet. (2003) 361:799–808. https://linkinghub.elsevier.com/retrieve/pii/S0140673603127055. (Accessed January 4, 2025) 12642045 10.1016/S0140-6736(03)12705-5

[4] Faedda GL Becker I Baroni A Tondo L Aspland E Koukopoulos A . The origins of electroconvulsive therapy: Prof. Bini’s first report on ECT. J Affect Disord. (2010) 120:12–5. doi: 10.1016/j.jad.2009.01.023 19268370

[5] Ottosson JO . Experimental studies of the mode of action of electroconvulsive therapy: Introduction. Acta Psychiatr Scand Suppl. (1960) 35:5–6. doi: 10.1111/j.1600-0447.1960.tb08347.x 14429446

[6] Bolwig TG Madsen TM . Electroconvulsive therapy in melancholia: The role of hippocampal neurogenesis. Acta Psychiatr Scand. (2007) 115:130–5. doi: 10.1111/j.1600-0447.2007.00971.x 17280579

[7] Bolwig TG . How does electroconvulsive therapy work? Theories on its mechanism. Can J Psychiatry. (2011) 56:13–8. doi: 10.1177/070674371105600104 21324238

[8] Sackeim H . The anticonvulsant hypothesis of the mechanisms of action of ECT: current status. J ECT. (1999) 15:5–26. doi: 10.1097/00124509-199903000-00003 10189616

[9] Sackeim HA Decina P Prohovnik I Malitz S Resor SR . Anticonvulsant and antidepressant properties of electroconvulsive therapy: a proposed mechanism of action. Biol Psychiatry. (1983) 18:1301–10. 6317065

[10] Ong MJY Lee VLL Teo SL Tan HJ Trinka E Khoo CS . Electroconvulsive therapy in refractory and super-refractory status epilepticus in adults: A scoping review. Neurocrit Care. (2024) 41:681–90. doi: 10.1007/s12028-024-02003-4 38769254

[11] Trinka E Cock H Hesdorffer D Rossetti AO Scheffer IE Shinnar S . A definition and classification of status epilepticus - Report of the ILAE Task Force on Classification of Status Epilepticus. Epilepsia. (2015) 56:1515–23. doi: 10.1111/epi.2015.56.issue-10 26336950

[12] Whittaker R Scott A Gardner M . The prevalence of prolonged cerebral seizures at the first treatment in a course of electroconvulsive therapy. J ECT. (2007) 23:11–3. doi: 10.1097/01.yct.0000263253.14044.3a 17435565

[13] Isenberg K Dinwiddie SH Song J North CS . A retrospective matched comparison study of prolonged seizures in ECT. J ECT. (2024) 40:37–40. doi: 10.1097/YCT.0000000000000951 37530874

[14] Hazimeh M Arnoudse N Wilson S Walczak T Nahas Z . Preliminary guidelines for resuming electroconvulsive therapy after a complication of status epilepticus. J ECT. (2024) 00:1–3. doi: 10.1097/YCT.0000000000001036 38975750

[15] Warren N Eyre-Watt B Pearson E O’Gorman C Watson E Lie D . Tardive seizures after electroconvulsive therapy. J ECT. (2022) 38:95–102. doi: 10.1097/YCT.0000000000000821 35093969

[16] Aftab A VanDercar A Alkhachroum A LaGrotta C Gao K . Nonconvulsive status epilepticus after electroconvulsive therapy: A review of literature. Psychosomatics. (2018) 59:36–46. doi: 10.1016/j.psym.2017.07.005 28802513

[17] Montgomery A Asberg M . Scale designed to be sensitive to change. Br J Psychiatry. (1979) 134:382–9. doi: 10.1192/bjp.134.4.382 444788

[18] Parker G McCraw S . The properties and utility of the CORE measure of melancholia. J Affect Disord. (2017) 207:128–35. doi: 10.1016/j.jad.2016.09.029 27721186

[19] Seymour J . Commentary and update on the contribution of the GABA hypothesis to understanding the mechanism of action of electroconvulsive therapy. J ECT. (2021) 37:4–9. doi: 10.1097/YCT.0000000000000711 32826706

[20] Duthie AC Perrin JS Bennett DM Currie J Reid IC . Anticonvulsant mechanisms of electroconvulsive therapy and relation to therapeutic efficacy. J ECT. (2015) 31:173–8. doi: 10.1097/YCT.0000000000000210 25621541

[21] Sanacora G Mason GF Rothman DL Hyder F Ciarcia JJ Ostroff RB . Increased cortical GABA concentrations in depressed patients receiving ECT. Am J Psychiatry. (2003) 160:577–9. doi: 10.1176/appi.ajp.160.3.577 12611844

[22] Esel E Kose K Hacimusalar Y Ozsoy S Kula M Candan Z . The effects of electroconvulsive therapy on GABAergic function in major depressive patients. J ECT. (2008) 24:224–8. doi: 10.1097/YCT.0b013e31815cbaa1 18562944

[23] Dalby NO Tønder N Wolby DPD West M Finsen B Bolwig TG . No loss of hippocampal hilar somatostatinergic neurons after repeated electroconvulsive shock: A combined stereological and in *situ* hybridization study. Biol Psychiatry. (1996) 40:54–60. doi: 10.1016/0006-3223(95)00355-X 8780855

[24] Shin HR Kim M Park KI . Electroconvulsive therapy and seizure: a double-edged sword? Encephalitis. (2023) 3:103–8. doi: 10.47936/encephalitis.2023.00059 PMC10598284 37621189

[25] Azuma H Fujita A Otsuki K Nakano Y Kamao T Nakamura C . Ictal electroencephalographic correlates of posttreatment neuropsychological changes in electroconvulsive therapy: A hypothesis-generation study. J ECT. (2007) 23:163–8. doi: 10.1097/YCT.0b013e31807a2a94 17804990

[26] Suppes T Webb A Carmody T Gordon E Gutierrez-Esteinou R Hudson JI . Is postictal electrical silence a predictor of response to electroconvulsive therapy? J Affect Disord. (1996) 41:55–8. doi: 10.1016/0165-0327(96)00066-3 8938205

[27] Zeiler FA Matuszczak M Teitelbaum J Gillman LM Kazina CJ . Electroconvulsive therapy for refractory status epilepticus: A systematic review. Seizure. (2016) 35:23–32. doi: 10.1016/j.seizure.2015.12.015 26789495

[28] Vignatelli L Tontini V Meletti S Camerlingo M Mazzoni S Giovannini G . Clinical practice guidelines on the management of status epilepticus in adults: A systematic review. Epilepsia. (2024) 65:1512–30. doi: 10.1111/epi.17982 38606469

[29] Akhtar SMM Saleem SZ Rizvi SHA Raja S Asghar MS . Beyond the surface: analyzing etomidate and propofol as anesthetic agents in electroconvulsive therapy—A systematic review and meta-analysis of seizure duration outcomes. Front Neurol. (2023) 14. doi: 10.3389/fneur.2023.1251882 PMC10616260 37915381

[30] Balki M Castro C Ananthanarayan C . Status epilepticus after electroconvulsive therapy in a pregnant patient. Int J Obstet Anesth. (2006) 15:325–8. doi: 10.1016/j.ijoa.2006.01.005 16774832

[31] Chathanchirayil SJ Bhat R . Post-electroconvulsive therapy status epilepticus and tardive seizure in a patient with rapid cycling bipolar disorder, epilepsy, and intellectual disability. J ECT. (2012) 28:183–4. doi: 10.1097/YCT.0b013e318248e1fb 22868490

[32] Conway CR Nelson LA . The combined use of bupropion, lithium, and venlafaxine during ECT: a case of prolonged seizure activity. J ECT. (2001) 17:216–8. doi: 10.1097/00124509-200109000-00014 11528316

[33] Crider BA Hansen-Grant S . Nonconvulsive status epilepticus as a cause for delayed emergence after electroconvulsive therapy. Anesthesiology. (1995) 82:591–3. doi: 10.1016/j.regsciurbeco.2008.06.005 7856921

[34] Dersch R Zwernemann S Voderholzer U . Partial status epilepticus after electroconvulsive therapy and medical treatment with bupropion. Pharmacopsychiatry. (2011) 44:344–6. doi: 10.1055/s-0031-1284425 21979924

[35] Devanand DP Decina P Sackeim HA Prudic J . Status Epilepticus following ECT in a Patient Receiving Theophylline. J Clin Psychopharmacol. (1988) 8:153. http://journals.lww.com/00004714-198804000-00027 (Accessed January 18, 2025). 3372714 10.1097/00004714-198804000-00027

[36] von Doellinger O Ribeiro JP Ribeiro Â Freitas C Ribeiro B Silva JC . Spontaneous seizures after ECT in a patient medicated with bupropion, sertraline and risperidone. Trends Psychiatry Psychother. (2016) 38:111–3. http://www.scielo.br/scielo.php?script=sci_arttext&pid=S2237-60892016000200111&lng=en&tlng=en. (Accessed January 18, 2025). 10.1590/2237-6089-2015-0055 27355895

[37] Goh SE Tor PC . Selecting right unilateral placement to facilitate continuation of electroconvulsive therapy following prolonged seizures. Asian J Psychiatr. (2021) 66:102874. doi: 10.1016/j.ajp.2021.102874 34624745

[38] Grogan R Wagner DR Sullivan T Labar D . Generalized nonconvulsive status epilepticus after electroconvulsive therapy. Convuls Ther. (1995) 11:51–6. doi: 10.1016/j.regsciurbeco.2008.06.005 7796069

[39] Jensen SS Christensen J Johnsen B Hjerrild S . Nonkonvulsiv status epilepticus efter elektrokonvulsiv terapi. Ugeskr Laeger. (2023) 185:1346–7. 36896614

[40] Jyoti Rao KM Gangadhar BN Janakiramaiah N . Nonconvulsive status epilepticus after the ninth electroconvulsive therapy. Convulsive Ther. (1993) 9:128–34. 11941202

[41] Katsumura T Okamoto N Tesen H Igata R Ikenouchi A Yoshimura R . Increased stimulation intensity helped to cope with prolonged seizures during the next round of modified electroconvulsive therapy: A case report. Int Med Case Rep J. (2022) 15:385–7. doi: 10.2147/IMCRJ.S374983 PMC9325873 35909591

[42] Kaufman KR Olsavsky A . Status epilepticus, electroconvulsive therapy and Malignant melanoma. Ir J Psychol Med. (2009) 26:87–9. doi: 10.1017/S0790966700000306 30282269

[43] Kramkowski J Rath S . Efficacious retrial of electroconvulsive therapy for major depressive disorder after a prolonged seizure in an older adult. BMJ Case Rep. (2023) 16:1–5. doi: 10.1136/bcr-2021-247633 PMC10163421 37130644

[44] Lang FU Klug R Lang S Walther B Jäger M . Convulsive status epilepticus after electroconvulsive therapy. German J Psychiatry. (2013) 16:81–3.

[45] Park HY Lee Y Kim D . Administration of electroconvulsive therapy with an anesthesia machine. J ECT. (2021) 37:e31–2. doi: 10.1097/YCT.0000000000000765 34048373

[46] Peters SG Wochos DN Peterson GC . Status epilepticus as a complication of concurrent electroconvulsive and theophylline therapy. Mayo Clin Proc. (1984) 59:568–70. doi: 10.1016/S0025-6196(12)61495-5 6748746

[47] Pogarell O Ehrentraut S Rüther T Mulert C Hegerl U Möller HJ . Prolonged confusional state following electroconvulsive therapy -Diagnostic clues from serial electroencephalography. Pharmacopsychiatry. (2005) 38:316–20. doi: 10.1055/s-2005-916187 16342004

[48] Povlsen UJ Wildschiødtz G Høgenhaven H Bolwig TG . Nonconvulsive status epilepticus after electroconvulsive therapy. J ECT. (2003) 19:164–9. doi: 10.1097/00124509-200309000-00009 12972987

[49] Reeve-Johnson L Alston Unwin HM . Generalised Non-Convulsive Status Epilepticus (NCSE) following Electro- Convulsive Therapy. J Psychol Psychother. (2014) 04:10–1. https://www.omicsonline.org/open-access/generalised-non-convulsive-status-epilepticus-NCSE-following-electro-convulsive-therapy-2161-0487.1000138.php?aid=24799 (Accessed January 26, 2025).

[50] Reti IM Davydow DS . Electroconvulsive therapy and antibiotics: A case report. J ECT. (2007) 23:289–90. doi: 10.1097/YCT.0b013e31813e06af 18090707

[51] Reyes-Molón L Trebbau-López H Saiz-González D . Epileptic status as a complication of electroconvulsive therapy: a case report. Actas Esp Psiquiatr. (2012) 40:99–101. 22508076

[52] Rucker J Cook M . A case of prolonged seizure after ect in a patient treated with clomipramine, lithium, l-tryptophan, quetiapine, and thyroxine for major depression. J ECT. (2008) 24:272–4. doi: 10.1097/YCT.0b013e31815bd768 18648320

[53] Scott AIF Riddle W . Status Epilepticus after Electroconvulsive Therapy. Br J Psychiatry. (1989) 155:119–21. https://www.cambridge.org/core/product/identifier/S0007125000176986/type/journal_article (Accessed January 18, 2025). 10.1192/bjp.155.1.119 2605416

[54] Shadman S Denyer R Owuor J Al-Mashat M . Status epilepticus following ect in an elderly patient: a case report and review of the literature. Chest. (2019) 156:A2247. doi: 10.1016/j.chest.2019.08.2166

[55] Solomons K Holliday S Illing M . Non-convulsive status epilepticus complicating electroconvulsive therapy. Int J Geriatric Psychiatry. (1998) 13:731–4. doi: 10.1002/(SICI)1099-1166(1998100)13:10<731::AID-GPS831>3.0.CO;2-L 9818310

[56] Srzich A Turbott J . Nonconvulsive generalised status epilepticus following electroconvulsive therapy. Aust New Z J Psychiatry. (2000) 34:334–6. doi: 10.1080/j.1440-1614.2000.00713.x 10789539

[57] Thisayakorn P Karim Y Yamada T McCormick LM . A case of atypical tardive seizure activity during an initial ECT titration series. J ECT. (2014) 30:77–80. doi: 10.1097/YCT.0b013e31829c10d6 23845940

[58] Varma NK Lee SI . Nonconvulsive status epilepticus following electroconvulsive therapy. Neurology. (1992) 42:263–3. doi: 10.1212/WNL.42.1.263 1734317

[59] Weiss JR Baker LP . Non-convulsive status epilepticus in a patient with schizoaffective and seizure disorder on clozapine and electroconvulsive therapy: A case report. Cureus. (2022) 450:3–6. doi: 10.7759/cureus.25337 PMC9232388 35761918

[60] Wieben E Kjeldsen MJ Sørensen CH . Convulsive status epilepticus induced by electroconvulsive therapy in a patient with major depression. Case Rep Psychiatry. (2022) 2022:2016–8. doi: 10.1155/2022/8545991 PMC8947920 35342656

[61] Weiner RD . ECT-induced status epilepticus and further ECT: a case report. Am J Psychiatry. (1981) 138:1237–8. doi: 10.1176/ajp.138.9.1237 7270732

[62] Prakash R Leavell SR . Status epilepticus with unilateral ECT: Case report. J Clin Psychiatry. (1984) 45:403–4. 6469928

[63] Kaufman KR Finstead BA Kaufman ER . Status epilepticus following electroconvulsive therapy. Mt Sinai J Med. (1986) 53:119–22. 3486347

[64] Chung KF . Relationships between seizure duration and seizure threshold and stimulus dosage at electroconvulsive therapy: Implications for electroconvulsive therapy practice. Psychiatry Clin Neurosci. (2002) 56:521–6. doi: 10.1046/j.1440-1819.2002.01048.x 12193241

[65] Girish K Gangadhar BN Janakiramaiah N . Merits of EEG monitoring during ect: a prospective study on 485 patients. Indian J Psychiatry. (2002) 44:24–248. 21206877 PMC2953649

[66] Gálvez V Hadzi-Pavlovic D Wark H Harper S Leyden J Loo CK . The anaesthetic-ECT time interval in electroconvulsive therapy practice - is it time to time? Brain Stimul. (2016) 9:72–7. doi: 10.1016/j.brs.2015.09.005 26452698

[67] Parsanoglu Z Balaban OD Gica S . Comparison of the clinical and treatment characteristics of patients undergoing electroconvulsive therapy for catatonia indication in the context of gender. Clinical EEG and Neuroscience (2022) 53(3):175–83. doi: 10.1177/15500594211025889 34142904

[68] Kranaster L Hoyer C Krisam M Deuschle M Janke C Sartorius A . Electroconvulsive therapy in a patient after radiation treatment of a brain metastasis: A case report. J ECT. (2012) 28:250–1. doi: 10.1097/YCT.0b013e318256ce29 22669038

[69] Nissen T Wynn R . The clinical case report: A review of its merits and limitations. BMC Res Notes. (2014) 7:1–7. doi: 10.1186/1756-0500-7-264 24758689 PMC4001358

[70] Fink M . Interseizure EEG slowing after ECT is not NCSE [1. Pharmacopsychiatry. (2006) 39:119. doi: 10.1055/s-2006-941490 16721705

[71] Fink M . Nonconvulsive status epilepticus and electroconvulsive therapy. J ECT. (2004) 20:131–2. http://journals.lww.com/00124509-200406000-00013 (Accessed January 6, 2025). 10.1097/00124509-200406000-00013 15167434

[72] Bolwig TG MDPovlsen UJM . Status epilepticus and ECT: A reply to dr. Fink. J Of Ect. (2004) 20:274–5. doi: 10.1097/00124509-200412000-00020 15591867

