# Supplementary material for: Restimulation could stop status epilepticus after electroconvulsive therapy: 2 case reports
Source: Front Psychiatry. 2025 May 29;16:1576374. doi: 10.3389/fpsyt.2025.1576374 (PMC12160024; doi:10.3389/fpsyt.2025.1576374)
Supplement: Supplementary file 1 [file Table1.docx]

Supplementary Material

## Search strategy for review of the literature to identify status epilepticus after ECT:

Inclusion criteria:

ECT performed within 1 week of seizure activity

Generalized seizure duration > 5min, partial or absence seizure duration >10min, continuous or intermittent, following ILAE classification(11)

Exclusion criteria

1. Not enough information about seizure duration
2. Not enough information to be certain of seizure activity

No language or time restrictions were applied.
We searched Pubmed with following query:
((ECT[Title/Abstract]) OR (electroconvulsive therapy[Title/Abstract]) OR (electro-convulsive therapy[Title/Abstract]) OR (convulsive therapy[Title/Abstract]) OR (electroshock[Title/Abstract)) AND ( (prolonged seizure*[Title/Abstract]) OR (status epilepticus[Title/Abstract]) OR (tardive seizure*[Title/Abstract]) OR (spontaneous seizure*[Title/Abstract]) )

This yielded 288 results. 7 more articles were included from the systematic review by Warren et al (15). 1 author (MP) screened 295 abstracts, of which 55 articles were sought for retrieval. 1 articles was not retrieved. 54 articles were evaluated for eligibility. Of these, 35 met inclusion criteria:

| Ref | Title | First Author | Year of publication |
| --- | --- | --- | --- |
| (30) | Status epilepticus after electroconvulsive therapy in a pregnant patient. | Balki | 2006 |
| (73) | Post–electroconvulsive therapy status epilepticus and tardive seizure in a patient with rapid cycling bipolar disorder, epilepsy, and intellectual disability | Chathanchirayil | 2012 |
| (32) | The combined use of bupropion, lithium, and venlafaxine during ECT: A case of prolonged seizure activity | Conway | 2001 |
| (33) | Nonconvulsive Status Epilepticus as a Cause for Delayed Emergence after Electroconvulsive Therapy | Crider | 1995 |
| (34) | Partial status epilepticus after electroconvulsive | Dersch | 2011 |
| (35) | Status epilepticus following ect in a patient receiving theophylline | Devanand | 1988 |
| (36) | Spontaneous seizures after ECT in a patient medicated with bupropion, sertraline and risperidone | Doellinger | 2016 |
| (37) | Selecting right unilateral placement to facilitate continuation of electroconvulsive therapy following prolonged seizures | Goh | 2021 |
| (38) | Generalized Nonconvulsive Status Epilepticus After Electroconvulsive Therapy | Grogan | 1995 |
| (14) | Preliminary Guidelines for Resuming Electroconvulsive Therapy after a Complication of Status Epilepticus | Hazimeh | 2024 |
| (39) | Nonkonvulsiv status epilepticus efter elektrokonvulsiv terapi | Jensen | 2023 |
| (40) | Nonconvulsive Status Epilepticus after the Ninth Electroconvulsive Therapy * | Jyoti Rao | 1993 |
| (41) | Increased Stimulation Intensity Helped to Cope with Prolonged Seizures During the Next Round of Modified Electroconvulsive Therapy: A Case Report | Katsumura | 2022 |
| (63) | Status Epilepticus following electroconvulsive therapy | Kaufman | 1986 |
| (42) | Status epilepticus, electroconvulsive therapy and malignant melanoma | Kaufman | 2009 |
| (43) | Efficacious retrial of electroconvulsive therapy for major depressive disorder after a prolonged seizure in an older adult | Kramkowski | 2023 |
| (44) ⴕ | Convulsive status epilepticus after electroconvulsive therapy | Lang | 2013 |
| (45) | Administration of Electroconvulsive Therapy With an Anesthesia Machine | Park | 2021 |
| (46) | Status epilepticus as a complication ofconcurrent electroconvulsive and theophylline therapy. | Peters | 1984 |
| (47) | Prolonged confusional state following electroconvulsive therapy -Diagnostic clues from serial electroencephalography | Pogarell | 2005 |
| (48) ⴕⴕ | Nonconvulsive status epilepticus after electroconvulsive therapy | Povlsen | 2003 |
| (62) | Status Epilepticus with unilateral ECT: case report | Prakash | 1984 |
| (49) | Generalised non-convulsive status epilepticus (NCSE) following electro-convulsive therapy | Reeve-Johnson | 2014 |
| (50) | Electroconvulsive therapy and antibiotics: A case report | Reti | 2007 |
| (51) | Epileptic status as a complication of electroconvulsive therapy: a case report. | Reyes-Molón | 2012 |
| (52) | A case of prolonged seizure after ECT in a patient treated with clomipramine, lithium, L-tryptophan, quetiapine, and thyroxine for major depression | Rucker | 2008 |
| (53) | Status Epilepticus after Electroconvulsive Therapy | Scott | 1989 |
| (54) | Status epilepticus following ECT in an elderly patient: a case report and review of the literature. | Shadman | 2019 |
| (55) | Non-convulsive status epilepticus complicating electroconvulsive therapy | Solomons | 1998 |
| (56) | Nonconvulsive generalised status epilepticus following electroconvulsive therapy | Srzich | 2000 |
| (57) | A case of atypical tardive seizure activity during an initial ECT titration series | Thisayakorn | 2014 |
| (58) | Nonconvulsive status epilepticus following electroconvulsive therapy | Varma | 1992 |
| (61) | ECT-induced status epilepticus and further ECT: A Case Report | Weiner | 1981 |
| (59) | Non-convulsive Status Epilepticus in a Patient With Schizoaffective and Seizure Disorder on Clozapine and Electroconvulsive Therapy: A Case Report | Weiss | 2022 |
| (60) | Convulsive Status Epilepticus Induced by Electroconvulsive Therapy in a Patient with Major Depression | Wieben | 2022 |
| (15) | Tardive Seizures after electroconvulsive therapy | Warren | 2022 |

ⴕ 1 of 3 events included

ⴕⴕWe noted comments by Fink (71) and Bolwig et al (72) and believe there is sufficient evidence for 2 of the cases to be considered NCSE

## CARE checklist

| Topic | Item | Checklist item description | Reported on line | |
| --- | --- | --- | --- | --- |
| Title | 1 | The diagnosis or interventions in this case report including “case report” | 1 | |
| Key Words | 2 | 2 to 5 key words that identify diagnoses or interventions in this case report, including "case report" | 12-13 | |
| Abstract (no refs) | 3a | What is unique about this case and what does it add to the scientific literature? | 26-29 | |
|  | 3b | Main symptoms and/or important clinical findings | 19-23 | |
|  | 3c | The main diagnoses, therapeutic interventions, and outcomes | 21-25 | |
|  | 3d | Conclusion—What is the main “take-away” lesson(s) from this case? | 26-29 | |
| Introduction | 4 | One or two paragraphs summarizing why this case is unique (may include references) | 30-55 | |
| Case presentation | | | Case A | Case B |
| Patient information | 5a | De-identified patient specific information | 59 | 94 |
|  | 5b | Primary concerns and symptoms of the patient | 65-66, | 99-101 |
|  | 5c | Medical, family, and psycho-social history including relevant genetic information | 59-61, | 94-97 |
|  | 5d | Relevant past interventions with outcomes . | 61-63, | 95-97 |
|  | 6 | Describe significant physical examination (PE) and important clinical findings | 65-68, 70-71 | 99-104 |
|  | 7 | Historical and current information from this episode of care organized as a timeline | Figure 1 | Figure 1 |
| Diagnostic assessment | 8a | Diagnostic testing (such as PE, laboratory testing, imaging, surveys). | 65-68 | 99-105 |
|  | 8b | Diagnostic challenges (such as access to testing, financial, or cultural) . | / | / |
|  | 8c | Diagnosis (including other diagnoses considered) | 67-68 | 101 |
|  | 8d | Prognosis (such as staging in oncology) where applicable | / | / |
| Therapeutic intervention | 9a | Types of therapeutic intervention (such as pharmacologic, surgical, preventive, self-care) | 69-70 | 101-106 |
|  | 9b | Administration of therapeutic intervention (such as dosage, strength, duration) . | 73-83 | 108-123 |
|  | 9c | Changes in therapeutic intervention (with rationale) | 78-83 | 118-123 |
| Follow-up and outcomes | 10a | Clinician and patient-assessed outcomes | 89-91 | 131-132 |
|  | 10b | Important follow-up diagnostic and other test results | 85-87 | 125-127 |
|  | 10c | Intervention adherence and tolerability (How was this assessed?) | 77-91 | 116-132 |
|  | 10d | Adverse and unanticipated events | 77-83 | 116-123 |
| Discussion | 11a | A scientific discussion of the strengths AND limitations associated with this case report | 153-209 | |
|  | 11b | Discussion of the relevant medical literature with references | 134-152, 166-192 | |
|  | 11c | The scientific rationale for any conclusions (including assessment of possible causes) | 133-192 | |
|  | 11d | The primary “take-away” lessons of this case report (without references) in a one paragraph conclusion | 210-217 | |
| Patient perspective | 12 | The patient should share their perspective in one to two paragraphs on the treatment(s) they received | Patients declined to comment | |
| Informed consent | 13 | Did the patient give informed consent? Please provide if requested | Yes, 475-480, supplementary materials | |

## Ethical approval and informed consent

Ethical approval was obtained from both the Ethical Committee (EC) of UZ Leuven and the EC of University Psychiatric Center KU Leuven, with study number S70079. Informed consent for this case report was obtained from both patients. For Case B, informed consent was given by both patient and a family member representing him.
